# Motion and Load Analysis of the Flexible Platform Based on Noncontact Processing

**DOI:** 10.3390/mi13070988

**Published:** 2022-06-24

**Authors:** Chao Lin, Mingdong Jiang, Ping Xu, Shan Zheng

**Affiliations:** 1 State Key Laboratory of Mechanical Transmission, Chongqing University, Chongqing 400030, China; 201907021036@cqu.edu.cn (M.J.); 201807131109@cqu.edu.cn (S.Z.); 2 Chongqing Panlian Transmission Technology Co., Ltd., Chongqing 400060, China; xuping_cq@126.com

**Keywords:** noncontact processing, compliant mechanism, stiffness, coupled displacement, motion trajectory

## Abstract

In this paper, we explore the applicability of the positioning stage based on flexible hinges for noncontact processing. According to the actual application of the positioning stage, Hooke’s law, the Euler–Bernoulli beam theory, and the geometric relationship of the structure are applied to analyze the coupled displacement in the movement of the positioning stage and the changes in the performance of the positioning stage caused by external loads. The coupled-displacement matrix and the external-load matrix obtained from the analysis are substituted into the ideal-displacement expression of the positioning stage to obtain the displacement expression of the platform in noncontact machining. The platform trajectory obtained by the referenced curve is analyzed. In addition, the coupled displacement in the X- and Y-directions and the coupled displacement caused by the external load in the Z-direction are nanoscales and about one-thousandth of the output displacement, which meets the requirement of tracking accuracy for micron-level machining. Finally, we use finite element analysis (FEA) and experiments to prove the correctness of the theoretical analysis.

## 1. Introduction

Under the action of the driving force, the compliant mechanism causes the elastic deformation of its flexible components and realizes the transmission of movement, force, and energy. Compared with the rigid mechanism, the outstanding advantage of the compliant mechanism is that it avoids friction, wear, and clearance and reduces vibration and noise. Moreover, the integrated processing does not require complicated assembly, especially nanolevel and high-precision movement. The most common flexible component is flexible hinges. Through the long-term research of related scholars, the forms of flexible hinges are rectangle-type [1,2], circular-type [3], vaulted-type [4], staggered-axis type [5], hybrid-type [6], and so on. Since the flexible component is slightly deformed, various flexible-displacement amplifiers have been invented to increase the moving stroke of the flexible mechanism. At present, the forms of flexible-displacement amplifier include bridge-type [7], rhombus-type [8,9], composite-type [10], Scott–Russell mechanism [11], lever-type [12], hybrid-type [13], and many more. The introduction of the flexible-displacement amplifier improves the moving stroke of the flexible mechanism and expands the applicable range of the flexible mechanism. Currently, in microgripper [14], ultraprecision machining [15], microvibration suppression [16], fluid transport and control [17], energy harvester [18], microelectromechanical systems [19], biomedicine [20], and other fields have used flexible mechanisms. Among them, the research on the precise positioning stage is more concentrated.

Table 1 compares the compliant micropositioning platforms developed based on flexible mechanisms. It can be seen from Table 1 that the current compliant micropositioning stage can realize multi-degree-of-freedom motion, the motion stroke is at the micrometer level, and there is a specific coupling motion. Among them, a guiding mechanism [21,22] is used to reduce the influence of coupling motion. Research on compliant micropositioning stages generally focuses on platform design [5,21,22], static/dynamic analysis [9,23], and kinematic analysis [2,24]. In order to promote the application of the platform, the motion control of the platform [1,25], error analysis [1,26], load analysis [27,28], and other aspects are studied. However, the application analysis of the platform is mainly for the two-dimensional motion platform and less for the six-degree-of-freedom motion platform. In addition, in the noncontact processing, the workpiece will be placed on the positioning stage, and the workpiece holder should be installed in advance, which will change the position and performance of the platform.

Therefore, this paper studies the motion and load of the flexible platform in non-contact machining. According to the practical application of the flexible platform, the analysis is carried out using Hooke’s law, Euler-Bernoulli beam theory and the geometric relationship of the structure. The motion performance changes of the flexible platform after the piezoelectric ceramic actuator (PZA) is installed, the displacement changes and coupled displacement changes of the flexible platform under load, and the coupled displacement changes in the Y/X direction caused by the movement in the X/Y direction are analyzed. In addition, the displacement expression of the flexible platform in non-contact machining is obtained through the derived mathematical equation. Finally, the correctness of the theoretical analysis and the feasibility of the flexible platform in non-contact machining is verified by simulation and experiment. It is worth mentioning that the crossed axis coupling displacement generated by parasitic motion when the platform only moves in the X/Y-direction is analyzed in the literature [33], and this coupling displacement is reduced to less than 1 μm through error compensation, which will not be studied in this paper. In this paper, the coupling displacement in the Y/X-direction caused by the movement of the platform in the X/Y-direction, and the coupling displacement in the Y/X-direction caused by the load in the Z-direction are analyzed.

## 2. Mechanism Description

**Figure 1:**
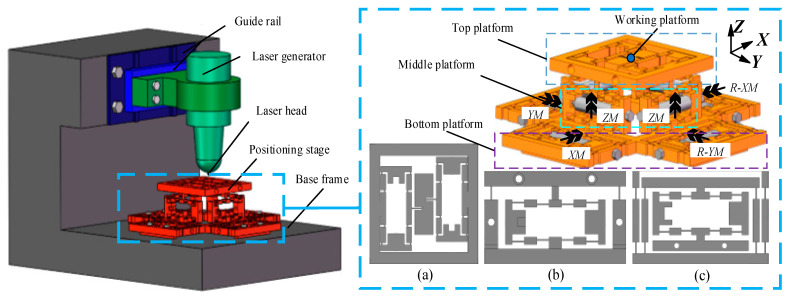
Three-dimensional solid modeling of laser machine and positioning stage. (**a**) The overlooking map of the top platform. (**b**)The main view of the middle platform. (**c**) A quarter view of the bottom platform.

By consulting the design manual of the laser machine and using SOLIDWORKS 2016 software (Solidworks, Concord, MA, USA) to perform a three-dimensional solid model of the laser machine, the solid model is shown in Figure 1. Different from the traditional laser machine, this device employs a flexible platform to achieve micron-level precision processing. Compared with the positioning stage of the transmission guide, the flexible platform achieves higher accuracy and is less affected by nonlinear factors, and it can better realize the processing of fine and small parts.

It can be seen from Figure 1 that the positioning stage is composed of the top platform, middle platform, and bottom platform. Figure 1a is the overlooking map of the top platform, Figure 1b is the main view of the middle platform, and Figure 1c is a quarter view of the bottom platform. The bottom platform realizes X-axis movement (*XM*), Y-axis movement (*YM*), X-axis reverse movement (*R*-*XM*), and Y-axis reverse movement (*R*-*YM*), respectively, and the middle platform achieves Z-axis movement (*ZM*).

In laser processing, the laser generator emits a laser beam through the laser head to the surface of the workpiece, with high laser energy to remove, melt the material, and change the surface performance of the workpiece. The Z-axis motion of the machine includes the large stroke of the sliding guide to facilitate the assembly of the workpiece. The flexible platform can also realize the Z-axis motion to meet the processing requirements. During machining, the laser head releases the laser beam, and the desired shape of the part is performed through the moving trajectory of the platform. It is worth mentioning that the flexible platform can realize six-degree-of-freedom motion, including linear motion in X-, Y-, and Z-directions and rotation around X-, Y-, and Z-axes. However, it is difficult to apply to precision trajectory due to the large-coupling effect and small-rotation-angle of the flexible platform around X-, Y-, and Z-axes.

**Figure 2:**
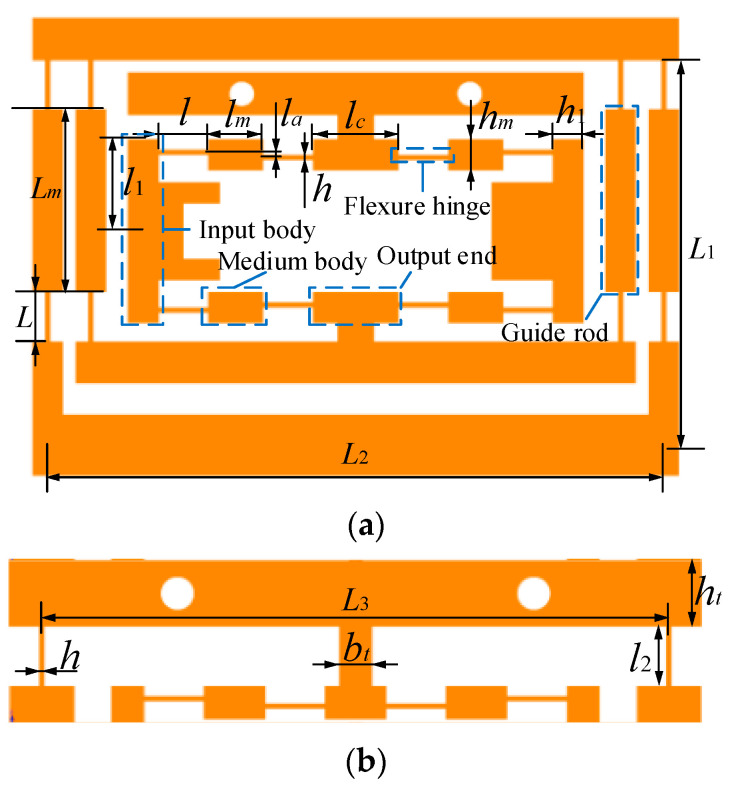
Schematic diagram of key parameters of the positioning platform. (**a**) Geometric parameter of the combined mechanism. (**b**) Geometric parameters related to the connection between the middle platform and the top platform.

For the rotation along with the X-, Y-, and Z-axis, there is a detailed analysis in [14]. The bridge-type displacement amplifier (BDA) with the same geometric parameters is employed in the positioning stage. Figure 1c is a quarter top view of the bottom platform and a combined mechanism of BDA and guiding mechanism. The relevant geometric parameter of the combined mechanism is depicted in Figure 2a, where the thickness of the combined mechanism is *b*. Figure 2b is the geometric parameters related to the joint between the middle and top platforms. Table 2 shows the parameter values in Figure 2 and platform materials.

## 3. The Kinematic Analysis of Positioning Stage

According to the research in the literature [9], the expression of the moving displacement *X_stg_* of the platform is as follows:

(1) $$X_{stg}=\frac{1}{1+\frac{R^{2}\cdot K_{out}\cdot K_{load}}{\left(K_{out}+K_{load}\right)\left(K_{p}+K_{in}\right)}}\cdot \frac{K_{out}}{K_{out}+K_{load}}\cdot \frac{K_{p}}{K_{p}+K_{in}}\cdot X_{p}\cdot R$$

where *R* is the displacement amplification ratio of BDA, *X_p_* is the output displacement of piezoelectric ceramic actuator (PZA), *K_p_* is the axial stiffness of PZA, *K_in_* is the input stiffness of BDA, *K_out_* is output stiffness of PZA, and *K_load_* is external-load stiffness of BDA.

In reference to [2], the calculating for the relevant parameters in Equation (1) is studied intensely. The calculating expression for the parameters in the literature [2] is as follows:

(2) $$R=\frac{I_{1}\left(\frac{l_{a}l^{2}}{4I}+\frac{E}{F_{x}}l_{a}\left(1-\cos\theta_{x}\right)+\frac{E}{F_{x}}l_{m}\sin\theta_{x}\right)-\frac{h_{1}^{2}}{240}\left(\mu +2+\frac{30l_{1}^{2}}{h_{1}^{2}}\right)-\frac{2F_{x}l_{1}^{2}}{K_{out}}\left(2+\frac{\left(8+9\mu \right)h_{1}^{2}}{20l_{1}^{2}}\right)}{\frac{EI_{1}}{F_{x}}\left(l_{a}-l_{z}\right)\sin\theta_{x}+\frac{2lI_{1}}{hb}+\frac{l_{m}I_{1}}{h_{m}b}+\frac{l_{1}^{3}}{6}\left[1+\frac{3h_{1}^{2}}{5l_{1}^{2}}\left(1+\frac{11\mu }{12}\right)-\frac{3l_{a}}{4l_{1}}\right]}$$

where *θ_x_* = *F_x_l_a_l*/(4*EI*), *I* = *h*^3^*b*/12, *I*_1_ = *h*_1_^3^*b*/12, *E* is Young’s modulus of the material, *F_x_* is the input force of BDA, *μ* is shear modulus, and *I* and *I*_1_ are the inertia moment of the flexible hinge and BDA input, respectively.

(3) $$K_{in}=\frac{1}{\frac{l_{a}{}^{2}\cdot l}{4EI}+\frac{l}{Ehb}+\frac{l_{m}}{Eh_{m}b}+\frac{l_{1}^{3}}{6EI_{1}}\left[1+\frac{3h_{1}^{2}}{5l_{1}^{2}}\left(1+\frac{11\mu }{12}\right)-\frac{3l_{a}}{4l_{1}}\right]}$$

(4) $$K_{out}=\frac{6EI}{4l^{3}+6l_{m}l^{2}+3l_{m}^{2}l}$$

The movement of the bottom platform will be hindered by the relative BDA and guiding mechanism. Therefore, the calculation of *K_load_* needs to obtain the output stiffness *K_out_* of the BDA and the swing stiffness *K_w_* of the guide mechanism. The expression of *K_w_* is as follows:

(5) $$K_{w}=\frac{12EI_{g}}{4L_{}^{3}+6L_{}^{2}L_{m}+3LL_{m}^{2}}$$

where *I_g_* is the inertia moment of the guiding mechanism.

The positioning stage platform needs the drive of PZA in the application, so the output stiffness of the BDA with PZA installed changes, and the output stiffness is no longer *K_out_* but *K_outb_*. Next, solve *K_outb_* in the platform.

**Figure 3:**
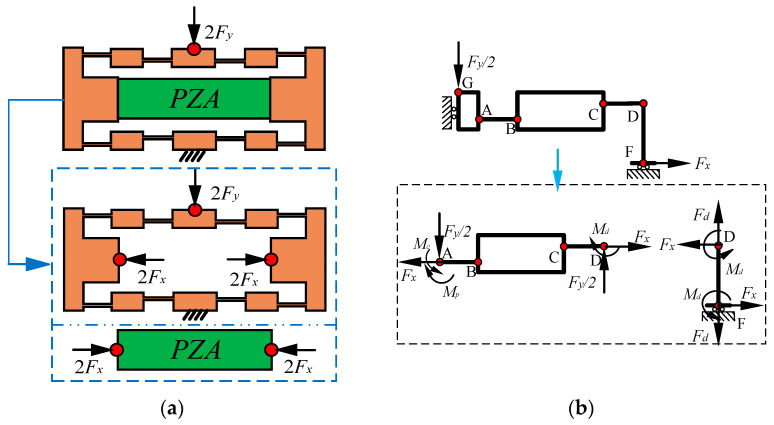
Force analysis of BDA equipped with PZA under the action of external load 2*F_y_*. (**a**) Force analysis of splitting BDA and PZA. (**b**) Force analysis at a quarter of BDA under the action of *F_y_*/2 and *F_x_*. *F_x_*, *F_y_* and *F_d_* are force vectors applied and *M_c_*, *M_d_* and *M_p_* are the moments.

When the external load 2*F_y_* acts on the BDA, the input body of the BDA driven by PZA will produce an interaction force 4*F_x_* to inhibit the deformation of the BDA. Hence, as shown in Figure 3a, when 2*F_y_* is applied to the output of BDA, BDA will be affected by 2*F_y_* and 4*F_x_*, and PZA will be squeezed by 4*F_x_*. Suppose that PZA is taken as the object of inhibiting movement for BDA, according to the force analysis at a quarter of BDA in Figure 3b. In that case, the displacement changes of BDA caused by the combined action of 4*F_x_* and 2*F_y_* includes:

- The total displacement *x_k_* of the input body of BDA without PZA is caused by the action of 2*F_y_*.
- When the PZA hinders the deformation of the BDA, an X-direction force 4*F_x_* is generated, which causes the input body of the BDA to produce a restrained displacement *x_f_*_._
- Under the action of 4*F_x_*, PZA produces compression displacement *x_y_*, and it is considered that *x_y_* is the final displacement of BDA in the X-direction under the action of 2*F_y_*.

Through the above analysis, the following relationships can be obtained as:

(6) $$x_{k}-x_{f}=x_{y}$$

Combining the analysis in literature [2] and Figure 3, the expression of each variate in Equation (6) is obtained, as shown below:

(7) $$\left\{ \begin{array}{l} x_{k}=\frac{F_{y}\left(2l+l_{m}\right)l_{1}^{2}}{4EI}+2\left(l_{a}-\frac{l_{m}\sin\left(\theta_{A}\right)}{\cos\left(\theta_{A}\right)}\right)\sin\left(\theta_{A}\right)\\ x_{f}=\frac{4F_{x}}{K_{in}}\\ x_{y}=\frac{2F_{x}}{K_{p}} \end{array}\right.$$

where *θ_A_* is the rotation angle of the end for flexible hinges under the action of *F_y_*/2 for the BDA without PZA, the *θ_A_* is derived as follows:

(8) $$\theta_{A}=\frac{F_{y}\left(l+l_{m}\right)l}{4EI}$$

Substituting Equation (7) into Equation (6) allows the generation of *F_x_*. Bring the obtained *F_x_* into *y_x_* = 4*F_x_R*/*K_in_* to get the restrained displacement *y_x_* of the BDA in the Y-direction. The output displacement *y_k_* of the BDA without PZA under the action of 2*F_y_* is reached by *y_k_* = 2*F_y_*/*K_out_*. From *y* = *y_k_*-*y_x_*, the actual displacement *y* of the output end for the BDA equipped with PZA under the action of 2*F_y_* is obtained. Then, *K_outb_* is expressed according to Hooke’s law, and its expression is as follows:

(9) $$K_{outb}=\frac{F_{y}}{2\left[\left(\frac{F_{y}l^{3}}{3EI}+\frac{F_{y}l_{m}l^{2}}{4EI}\right)+l_{a}\left(1-\cos\theta_{A}\right)+l_{m}\cdot \sin\theta_{A}\right]-\frac{4RF_{x}}{K_{in}}}$$

**Figure 4:**
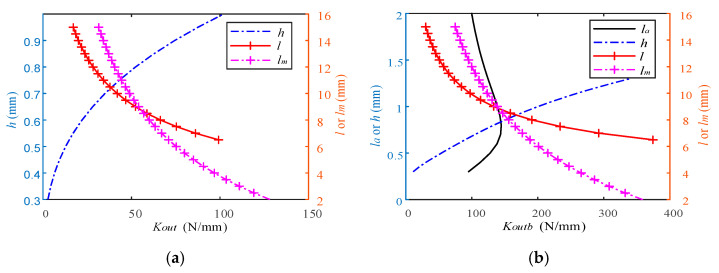
The relationship between geometric parameters and stiffness *K_outb_* and *K_out_* in BDA. (**a**) The relationship between geometric parameters and *K_outb_*. (**b**) The relationship between geometric parameters and *K_out_*.

Combining Equations (1)–(5) and (9), the output displacement of the platform in the X- and Y-directions under the action of different PZA numbers can be obtained. In order to observe the influence of PZA on the output stiffness of BDA, the relationship between the crucial parameters in BDA and its output stiffness is analyzed. Figure 4 shows the relationship between crucial parameters in BDA and *K_outb_* and *K_out_*, respectively. As shown in Figure 4, the output stiffness of BDA is significantly increased after PZA is installed. Among them, *l_a_* has a relationship with *K_outb_* but has nothing to do with *K_out_*. This is because *y_k_* has nothing to do with *l_a_*, and *y_x_* is related to *l_a_*, so there is a relationship between *y* and *l_a_* to get the relationship between *K_outb_* and *l_a_*.

## 4. The Load Analysis of Positioning Stage

The positioning stage may be affected by the Z-direction load in the application. For example, the platform is applied to the microscope to fine-tune the position of the sample. At this time, the top of the positioning platform will be affected by the gravitational load of the sample and the fixed sample device. Therefore, it is necessary to analyze the influence of the positioning platform on the Z-direction load.

**Figure 5:**
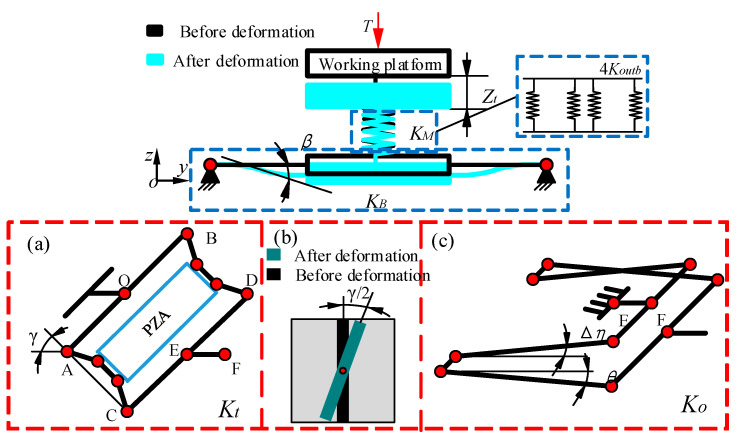
The impact of external load in Z-direction on the platform. (**a**) BDA deformation diagram. (**b**) Cross-section of torsion of the single flexible hinge. (**c**) Deformation diagram of guiding mechanism. *T* is force vector applied. *K_M_* is the stiffness of the middle platform in the Z-direction. *K_B_* is the stiffness of the bottom platform in the Z-direction. *Kt* is the roll stiffness for BDA in the Z-direction. *K_o_* is the Z-direction roll stiffness of the guide mechanism in the bottom platform. *K_outb_* is output stiffness of BDA with PZA. *Z_t_* is the Z-direction change displacement of the platform under the action of the external load *T*. *β*, *γ*, Δ*η* and *θ* are the roll angle of the combined mechanism, the roll angle of the BDA, and the roll angle of the two guide mechanisms, respectively.

First, the total displacement change in the Z-direction of the platform is being analyzed. As shown in Figure 5, under the action of the external load *T*, the middle platform, and the bottom platform descend simultaneously, and the total descending displacement is *Z_t_*. The four BDAs of the middle platform obstruct the load *T* to cause the platform to deform in the Z-direction. The combined mechanism formed by the BDA and the guiding mechanism in the bottom platform prevents the load *T* from causing the platform to deform in the Z-direction, and its roll angle is *β*. Since the Z-direction stiffness *K_M_* of the middle platform and the Z-direction stiffness *K_B_* of the bottom platform are in series, the expression of *Z_t_* is as follows:

(10) $$Z_{t}=\frac{T\left(K_{M}+K_{B}\right)}{K_{M}\cdot K_{B}}$$

where *K_M_* = 4*K_outb_* and *K_B_* is a series of *K_t_* and *K_o_*. *K_t_* is the roll stiffness for BDA in the Z-direction, and *K_o_* is the Z-direction roll stiffness of the guide mechanism in the bottom platform.

Under the action of load *T*, the X-, Y-, and Z-direction movement of the platform will change. For the Z-direction movement of the middle platform, it is necessary to overcome the effect of the external load *T* to make the platform rise. The X- and Y-direction movement is different from the Z-direction movement. Under the action of the load *T*, each combined mechanism of the BDA and the guiding mechanism in the bottom platform produces the same deformation, so only one combined mechanism can be studied. Figure 5a illustrates that the flexible hinges in the BDA are twisted under the influence of the torque, and its total torsion angle is *γ*. Figure 5b is a cross-sectional view of the torsion of a flexible hinge in BDA. The flexible hinges of the two guiding mechanisms are affected by the load *T* to produce bending deformation for the two guiding mechanisms. As a result, the medium body between the flexible hinges rotates with the flexible hinges. The tilt angles of the two guiding mechanisms are shown in Figure 5c as Δ*η* and *θ*, respectively.

**Figure 6:**
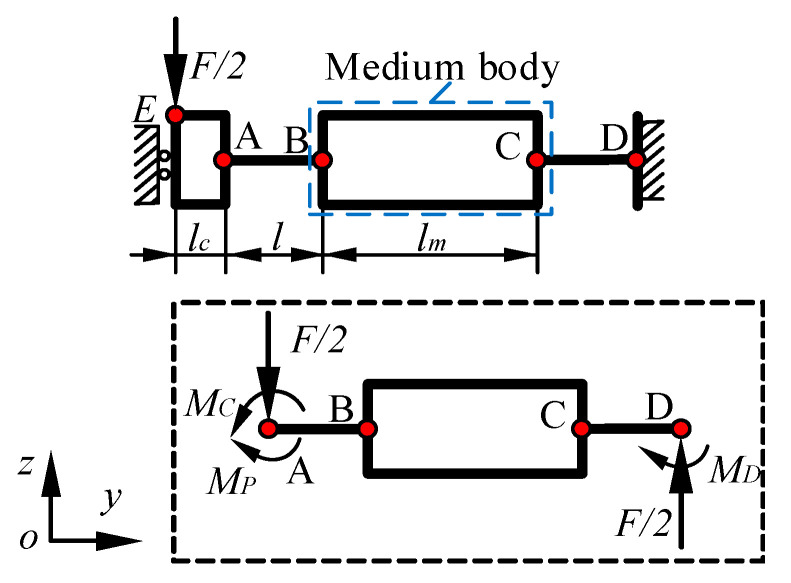
Schematic diagram of the flexible mechanism and its force analysis diagram. *F/2* is force vector applied and *M_C_*, *M_D_* and *M_P_* are the moments. *l_c_* is the lengths of the output end. *l* is the lengths of the flexure hinge. *l_m_* is the length of the medium body.

Since the combined mechanism formed by the BDA and the guiding mechanism in the bottom platform includes three groups of flexible mechanisms, a simplified diagram of the mechanism is depicted in Figure 6. It is assumed that only the flexible hinges are deformed, and the flexible hinge is subjected to the same load. Thus, it is considered that *M_B_* = *M_C_* in Figure 6. Combined with the force analysis in Figure 6, the following expressions can be generated as:

(11) $$\left\{ \begin{array}{l} M_{B}=M_{C}=\frac{Fl_{m}}{4}\\ M_{D}=\frac{F\left(2l+l_{m}\right)}{4}\\ M_{p}=\frac{F\left(2l+l_{m}+l_{c}\right)}{4} \end{array}\right.$$

According to Euler-Bernoulli beam theory, the calculating expression of the rotation angle *θ_B_* and the deflection *W_B_* at point B are respectively expressed as:

(12) $$\left\{ \begin{array}{l} \theta_{B}=\frac{F\left(l+l_{m}\right)l}{4EI}\\ W_{B}=\frac{Fl^{3}}{6EI}+\frac{Fl_{m}l^{2}}{8EI} \end{array}\right.$$

Due to the flexible hinges AB and CD being subjected to equivalent loads, the rotation angle and deflection of point C are the same as those at point B. Since the rotation angle of the medium body and the end of the flexible hinge in the flexible mechanism is equal, the maximum displacement *W_m_* of the medium body in the Z-direction changes to *l_m_*sin*θ_B_*. Combined with the displacement change of the flexible hinge, the displacement change at point E is *W_m_* + 2*W_B_*.

When the moment *M* is applied to point E, the analysis method is the same as the above, and the expression of the displacement change at point E is as follows:

(13) $$W_{T2}=\frac{Ml\left(l_{m}+l\right)}{2EI}$$

When the flexible hinge only produces torsion deformation, the analysis method is the same as the above. Take the torque *M* at the output end of BDA as an example. The displacement of the flexible hinge is small when it is twisted, but it drives the displacement of the input body to increase the displacement of the output end. The maximum displacement change *W_T_*_3_ of the input body is considered the total displacement change. This displacement expression can be written as:

(14) $$W_{T3}=\frac{Ml_{m}l}{2Gshb^{3}}$$

where *G* is the shear modulus and *s* is a factor related to the side length ratio of the section, which is taken as 1/3 in this article.

Based on the previous analysis, the displacement expression of the bottom platform under the load *T* in the Z-direction is taken as:

(15) $$W=\frac{TL\left[12L_{m}L+8L^{2}+6L_{m}{}^{2}+3L_{1}\left(L+L_{m}\right)\right]}{96EI_{2}}+\frac{TL_{1}ll_{1}}{32sGhb^{3}}+\frac{TL_{2}^{3}}{96EI_{3}}$$

Therefore, the Z-direction stiffness *K_B_* of the bottom platform is expressed as:

(16) $$K_{B}=1/(\frac{L\left[12L_{m}L+8L^{2}+6L_{m}{}^{2}+3L_{1}\left(L+L_{m}\right)\right]}{96EI_{2}}+\frac{L_{1}ll_{1}}{32sGhb^{3}}+\frac{L_{2}^{3}}{96EI_{3}})$$

## 5. The Analysis of Coupled Movement

In this paper, the positioning stage is a symmetrical structure, and a guiding mechanism is used to eliminate the cross-coupling between the axes of the bottom platform. The guide mechanism greatly eliminates cross-coupling between the X-axis and Y-axis. The remaining cross-coupling is affected by the symmetry of the positioning stage, causing the positioning stage to deform irregularly, which results in the cross-coupling displacements between axes on the upper surface of the top platform being unequal. Since the bottom platform and the middle platform are not on the same level, the cross-coupling displacement between X/Y-axis and Z-axis is minimal, mainly caused by the irregular deformation under the influence of the symmetry of the positioning stage. To sum up, the analysis of the cross-coupling between the axes of the positioning stage is complex, and the coupling displacement varies from place to place, so it will not be studied in the following. This section mainly studies the X/Y-direction movement causes coupled displacement in Y/X-direction, and the coupled displacement in the X- and Y-directions under load.

### 5.1. The X/Y-Direction Movement of Platform Causes Coupled Displacement in the Y/X-Direction

**Figure 7:**
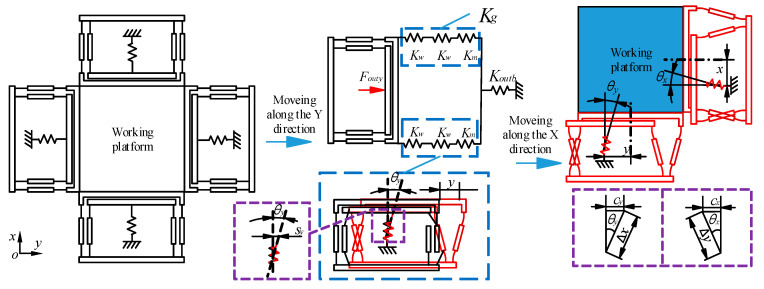
The model diagram of the influence of platform X/Y-axis motion on Y/X-axis motion.

Figure 7 is the moving analysis diagram of the platform, which is the influence of the X/Y-direction movement on the Y/X-direction movement. In the picture, the Y-direction movement of the platform starts, and the input force *F_outy_* in the Y-direction movement is generated under the actuation of the BDA as the input source. Under the action of the force *F_outy_*, the BDAs on the Y-axis produce linear movement, while the BDAs on the X-direction produce lateral movement. The stiffness *K_g_* of the X-direction combined mechanism for lateral movement is the series connection of the stiffness 2*K_w_* of the two guide mechanisms and the lateral stiffness *K_m_* of the BDA. The combined mechanism on the X-axis will produce a Y-direction displacement Δ*y*, and BDA produces not only an X-direction tilt angle *θ_y_* but also a Y-direction roll displacement *s_y_*.

When the Y-direction movement is completed, the X-direction movement is started. The X-direction movement is the same as the Y-direction movement. However, due to the Y-direction movement, the output end of the X-direction movement BDA produces a tilt angle *θ_y_*, which makes the Y-direction produce a coupled displacement *C_y_* caused by the X-direction displacement Δ*x*, and the X-direction will also exist the coupled displacement *C_x_* produced by the Y-direction displacement Δ*y*. Through the above analysis, the expression of the coupled displacement *C_y/x_* generated as:

(17) $$C_{y/x}=\frac{\Delta yK_{m}K_{w}}{2l_{1}\left(2K_{m}+K_{w}\right)K_{m}}\Delta x$$

The above expression can be simplified as *C_y/x_* = *A*Δ*x*Δ*y*, where *A* is the coupled constant of the platform X/Y-direction movement.

From Equation (17), it can be demonstrated that reducing the stiffness *K_g_*, increasing the lateral stiffness *K_m_* and width 2*l*_1_ of the BDA can reduce the coupled displacement *C_y/x_*. Meanwhile, *l*_1_ is related to BDA performance, so under the premise of considering BDA performance, *l*_1_ can be appropriately increased to reduce *C_y/x_*. Because the analysis of *K_m_* is complex, this article replaces it with simulated values.

### 5.2. The Z-Direction Load Causes Coupled Displacement in the Y/X-Direction

**Figure 8:**
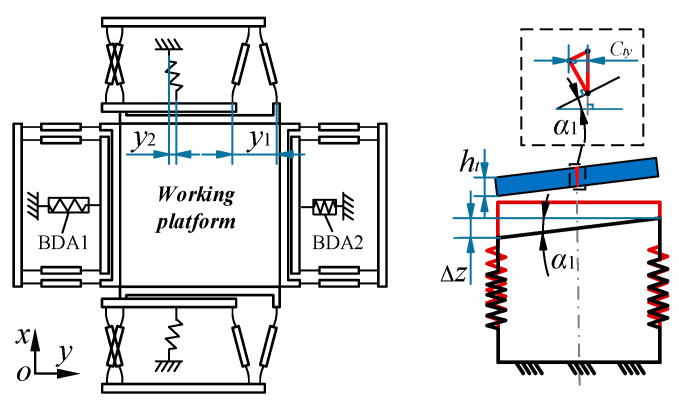
Coupled movement analysis in the Y-direction for the platform under load *T*.

After analyzing the displacement change of the platform in the Z-direction under the load *T*, the coupled displacement caused by the load *T* is then analyzed. When there is no movement in the X/Y-direction, the X- and Y-directions of the platform will not produce coupled displacement under the influence of the load *T*. When the platform produces displacement in the X/Y-direction, the original balance of the X- and Y-directions is broken to produce a coupled displacement. As shown in Figure 8, when the platform moves in the Y-direction, BDA1 and BDA2 on the Y-axis are respectively pulled and pressed, and the output displacements of the two BDAs change oppositely, where the output displacement of BDA1 increases by *y*_1_+*y*_2_. Under the action of the load *T*, the two BDAs will change, the BDA under tension will receive a more significant moment, and the BDA under compression will receive a little moment. Through the above analysis, the displacement of the BDA1 in Z-direction is reduced by ΔZ/2, the displacement of the BDA2 in Z-direction is increased by ΔZ/2, the top platform produces a tilt angle *α*_1_, and under the influence of the height *h_t_* of the top platform, the platform produces coupled displacement *C_ty_* in Y-direction.

Through the above analysis and according to the geometric relationship in Figure 8, the expression of the coupled displacement *C_tx/y_* in the X/Y-direction under the action of the load *T* can be obtained as follows:

(18) $$\left\{ \begin{array}{l} C_{tx}=2\sin\left(\frac{TL_{1}l}{64Gshb^{3}}\right)\frac{h_{t}\Delta x}{L_{3}}\\ C_{ty}=2\sin\left(\frac{TL_{1}l}{64Gshb^{3}}\right)\frac{h_{t}\Delta y}{L_{3}} \end{array}\right.$$

The Equation (18) can be simplified as *C_tx/y_* = *B*Δ*x*/*y*, where *B* is the coupled constant of X- and Y-direction movement under the load in the Z-direction.

Through Equation (18), it can be derived that the coupled displacement *C_tx/y_* can be reduced by appropriately reducing the thickness *h_t_* of the top platform, the length *l* of the flexible hinge for BDA in the bottom platform, and the total length *L*_1_ of the flexible hinge and the medium body in the guiding mechanism as well as increasing the thickness *b* and height *h* of the flexible hinge for BDA in the bottom platform and the side length *L*_3_ of the top platform. However, *b*, *h*, and *l* are related to the BDA of the input source and can be increased or decreased appropriately only under the premise of ensuring the performance of the BDA. *L*_3_ is closely related to the overall space of the platform and can be appropriately increased according to the place of application. The simple way to change the platform coupled displacement *C_tx/y_* is to reduce *h_t_* and *L*_1_.

### 5.3. The Pose Equation of Positioning Stage

Since the load stiffness exists when the platform moves in the X- and Y-directions, the displacement expression is in Equation (1). The Z-direction motion of the platform does not affect the load stiffness. When the load stiffness *K_load_* in Equation (1) is zero, it is the Z-direction motion equation of the platform. Through the above analysis, it is considered that the ideal motion expression of the platform without considering the coupling motion of the platform and the influence of the external load is as follows:

(19) $$\left\{ \begin{array}{l} S_{x}=W\cdot \frac{K_{out}}{K_{out}+K_{load}}\cdot \frac{K_{p}}{K_{p}+K_{in}}\cdot R\cdot X_{x}\\ S_{y}=W\cdot \frac{K_{out}}{K_{out}+K_{load}}\cdot \frac{K_{p}}{K_{p}+K_{in}}\cdot R\cdot X_{y}\Rightarrow \left(\begin{array}{l} S_{x}\\ S_{y}\\ S_{z} \end{array}\right)\\ S_{z}=\frac{K_{p}}{K_{p}+K_{in}}\cdot R\cdot X_{z} \end{array}\right.=\left(\begin{array}{l} \begin{array}{ccc} C & 0 & 0 \end{array}\\ \begin{array}{ccc} 0 & C & 0 \end{array}\\ \begin{array}{ccc} 0 & 0 & P \end{array} \end{array}\right)\left(\begin{array}{l} X_{x}\\ X_{y}\\ X_{z} \end{array}\right)$$

Equation (18) can be simplified as ***S*** = ***MX*** where ***S*** is the ideal output displacement of the platform, ***M*** is the ideal displacement matrix of the platform, and ***X*** is the input displacement of the platform.

Since the PZA is taken as the input source in the motion of the platform, the calculating expression of the input displacement for the positioning stage can be obtained according to the inverse position solution of the positioning stage:

(20) $$\left(\begin{array}{l} X_{x}\\ X_{y}\\ X_{z} \end{array}\right)=\left(\begin{array}{l} \begin{array}{ccc} 1/C & & \end{array}\\ \begin{array}{ccc} & 1/C & \end{array}\\ \begin{array}{ccc} & & 1/P \end{array} \end{array}\right)\left(\begin{array}{l} S_{x}\\ S_{y}\\ S_{z} \end{array}\right)$$

Convert the Equation (17) of the coupled displacement *C_y/x_* caused by the platform X/Y-axis movement to the Y/X-axis movement into a matrix expression and combine with Equation (19) to obtain the following expression:

(21) $$\left(\begin{array}{l} S_{cx}\\ S_{cy}\\ S_{cz} \end{array}\right)=\left(\begin{array}{l} \begin{array}{ccc} A & 0 & 0 \end{array}\\ \begin{array}{ccc} 0 & A & 0 \end{array}\\ \begin{array}{ccc} 0 & 0 & 0 \end{array} \end{array}\right)\left(\begin{array}{l} \begin{array}{ccc} S_{y} & 0 & 0 \end{array}\\ \begin{array}{ccc} 0 & S_{x} & 0 \end{array}\\ \begin{array}{ccc} 0 & {}_{}0 & 0 \end{array} \end{array}\right)\left(\begin{array}{l} S_{x}\\ S_{y}\\ S_{z} \end{array}\right)$$

Equation (20) can be simplified as ***S_c_*** = ***M*_1_*S*_1_*S***, where ***M*_1_** is the coupling displacement matrix of X- and Y-direction motions and ***S*_1_** is the output displacement matrix under the coupled action. Equation (18) of the coupled displacement *C_tx/y_* for the X/Y-direction motion under the action of the load *T* is transformed into a matrix expression, and the following expression is generated as:

(22) $$\left(\begin{array}{l} S_{tx}\\ S_{ty}\\ S_{tz} \end{array}\right)=\left(\begin{array}{l} \begin{array}{ccc} B & 0 & 0 \end{array}\\ \begin{array}{ccc} 0 & B & 0 \end{array}\\ \begin{array}{ccc} 0 & 0 & 0 \end{array} \end{array}\right)\left(\begin{array}{l} S_{x}\\ S_{y}\\ S_{z} \end{array}\right)$$

Equation (22) can be simplified as ***S_t_*** = ***M*_2_*S***, where ***M*_2_** is the coupled displacement matrix of X- and Y-direction motion caused by load *T*. The total coupled displacement matrix is the difference between ***S_c_*** and ***S_t_***. Based on Equations (19)–(22), the expression of the platform motion considering the influence of coupling displacement and Z-direction load is as follows:

(23) $$\left(\begin{array}{l} S_{sx}\\ S_{sy}\\ S_{sz} \end{array}\right)=\left(\begin{array}{l} S_{tx}\\ S_{ty}\\ S_{tz} \end{array}\right)-\left(\begin{array}{l} S_{cx}\\ S_{cy}\\ S_{cz} \end{array}\right)-\left(\begin{array}{l} 0\\ 0\\ W \end{array}\right)+\left(\begin{array}{l} S_{x}\\ S_{y}\\ S_{z} \end{array}\right)$$

Equation (23) can be simplified as ***S_s_*** = ***S_c_*** − ***S_t_*** − ***C***+***S***, where ***C*** is the Z-direction coupled constant caused by the load *T*.

## 6. Comparative Analysis of Theory, Experiment and Simulation

**Figure 9:**
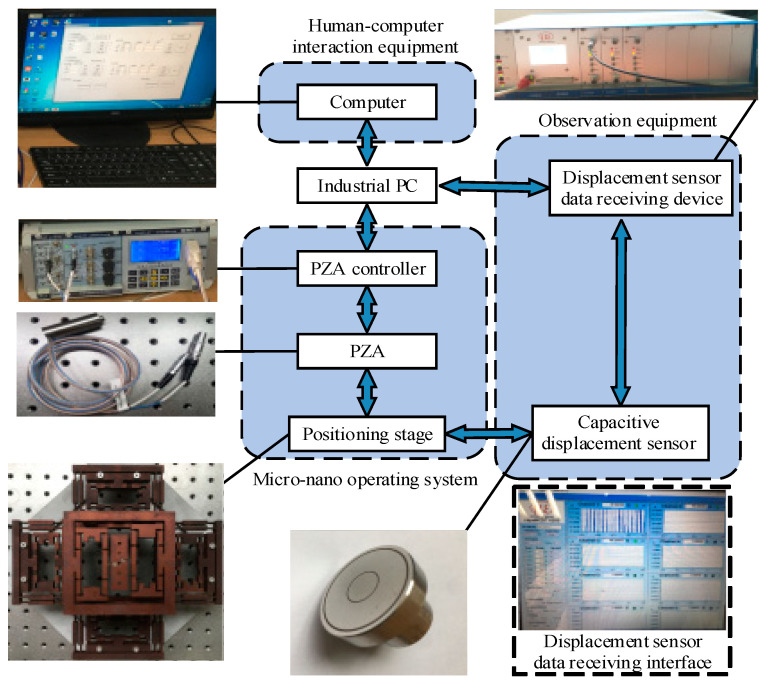
The experimental flowchart.

**Figure 10:**
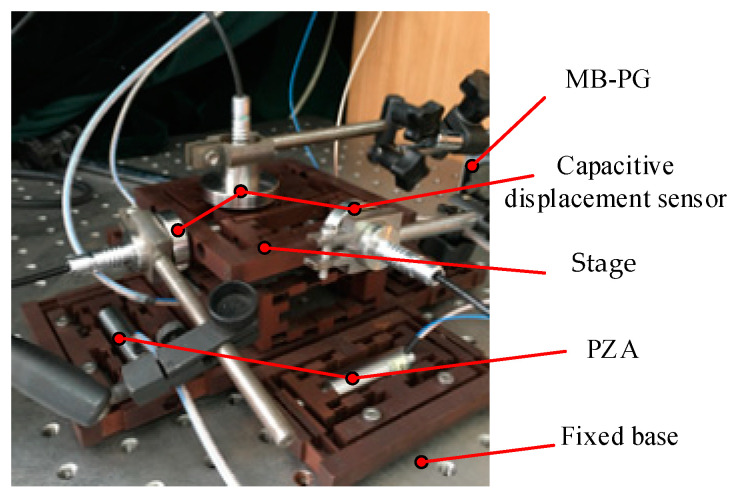
The layout of the positioning stage.

The positioning stage is driven by the PZA (model PSt-40VS15). The characteristics of PZA are shown in Table 3, and the maximum output displacement under 120 V driving voltage is 38 μm. When the PZA is installed in the BDA, it is fixed by bolts. Among them, the PZA controller can realize closed-loop control and eliminate the nonlinear error of PZA. A capacitive displacement sensor CS5 with a measurement range of 5 mm, a static resolution of 3.75 nm and a dynamic resolution of 100 nm is used to measure the output displacement of the platform. Finally, the measurement results are displayed on the computer. The experimental process is shown in Figure 9. The layout of the positioning stage in the experiment is shown in Figure 10.

In addition, due to the influence of machining accuracy, compared with theoretical and simulation analysis, there are errors in the geometric parameters of the positioning stage in the experiment, which leads to deviations between the experimental model and the theoretical and simulation models. Therefore, it is necessary to control and compensate for the machining error of the positioning stage in the experiment. Researchers [34] discuss the machining error of the positioning stage in this paper and use the drive displacement of the PZA to compensate for the machining error of the positioning stage. Since the positioning stage can realize X-, Y-direction reciprocating motion and Z-direction unidirectional motion, it is necessary to perform displacement compensation for the five-direction motion. According to Equation (20), the inverse equation of the platform motion with the displacement compensation coefficient added is obtained, which is shown by the following formula:

(24) $$\left(\begin{array}{l} X_{xr}\\ X_{xl}\\ X_{yr}\\ X_{yl}\\ X_{z} \end{array}\right)=\left[\begin{array}{lllll} \varepsilon_{xr} & & & & \\ & \varepsilon_{xl} & & & \\ & & \varepsilon_{yr} & & \\ & & & \varepsilon_{yl} & \\ & & & & \varepsilon_{z} \end{array}\right]\left[\begin{array}{lllll} C^{-1} & & & & \\ & C^{-1} & & & \\ & & C^{-1} & & \\ & & & C^{-1} & \\ & & & & P^{-1} \end{array}\right]\left(\begin{array}{l} S_{xr}\\ S_{xl}\\ S_{yr}\\ S_{yl}\\ S_{z} \end{array}\right)$$

where *ε* is the compensation coefficient for input displacement, *xr*, *xl*, *yr*, and *yl* are X-axis movement, X-axis reverse movement, Y-axis movement, and Y-axis reverse movement, respectively.

By measuring the surface of the positioning stage, the machining errors of various geometric parameters are obtained. Then the machining error is introduced into the motion equation of the positioning stage to obtain the motion equation after considering the machining error. Finally, the results of the two motion equations are compared, and the compensation coefficient for input displacement in each direction is obtained. After calculation, the compensation coefficient for input displacement in each direction [*ε_xr_*, *ε_xl_*, *ε_yr_*, *ε_yl_*, *ε_z_*] is [1.0274, 1.0257, 1.0287, 1.0234, 1.0226].

**Figure 11:**
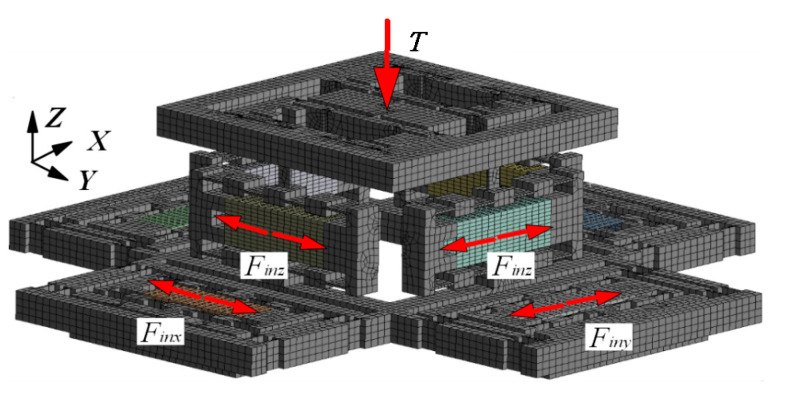
Finite element model.

For FEA, ANSYS 17 software (ANSYS, Inc., Canonsburg, PA, USA) is used for static analysis. The mesh is divided according to the hex dominant in the analysis, and the convergence is set to 5%. The finite element model is shown in Figure 11. In FEA, the relationship between the input and output displacement in the motion of each direction of the platform is first obtained. Then the input displacement of PZA to get the ideal output displacement is input into the simulation analysis to obtain the simulated output displacement. Finally, the simulated trajectory is obtained by the method of connecting points. For the theoretical analysis, the input displacement in FEA is used to obtain the theoretical trajectory of the platform.

**Figure 12:**
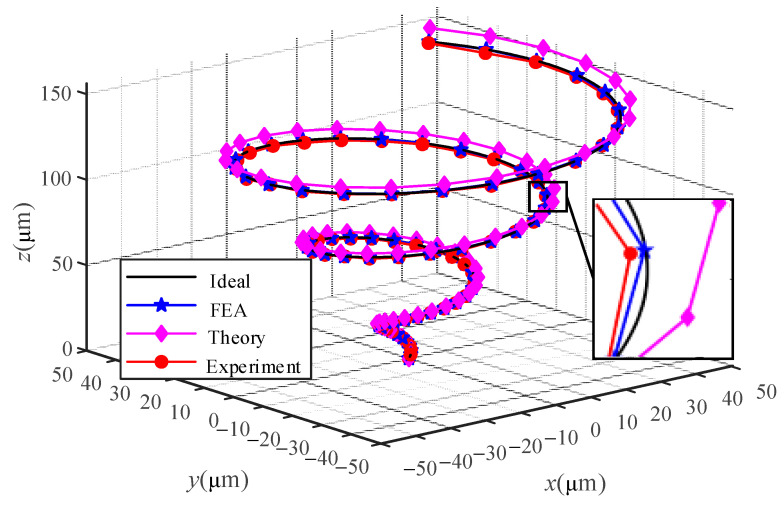
The comparison of conical spiral trajectory.

In the research, the conical spiral trajectory is used as the reference trajectory, and the experimental, simulated, and theoretical trajectory curves are obtained, respectively. Figure 12 is a comparison of conical spiral trajectories. It can be seen from Figure 12 that the experimental, simulation, and theoretical trajectories have fixed errors caused by the point-to-point connection method compared with the ideal trajectory. The FEA results and the experimental results are almost in agreement with the ideal values from the track points studied, while the theoretical results are close to the ideal values. Table 4 lists some of the values in the trajectory to better understand the data in the trajectory. Since the high accuracy of the FEA results is considered to be the actual value of the positioning stage, the experimental and theoretical errors in Table 4 are obtained based on the FEA results. It can be deduced from Table 4 that the error of the theoretical value relative to the simulated value tends to be a constant value, in which the error of the Z-direction movement is about 4.12%, and the error of the X-direction movement is about 5.25%. The large deviation of the theoretical value from the simulated value may be due to: (1) In the theoretical modeling, the complex deformation of the positioning stage is simplified. (2) In the theoretical modeling, the influence of the self-weight of the positioning stage on its performance is ignored. The error of the experimental value relative to the simulated value is variable, which may be due to the measurement noise, coupling errors, etc. The error of the Z- and X-direction movement in the experiment is less than 1.5%. The above analysis shows that the correctness of the equation of motion and the correctness of the calculation of the relevant parameters in the equation can be found.

**Figure 13:**
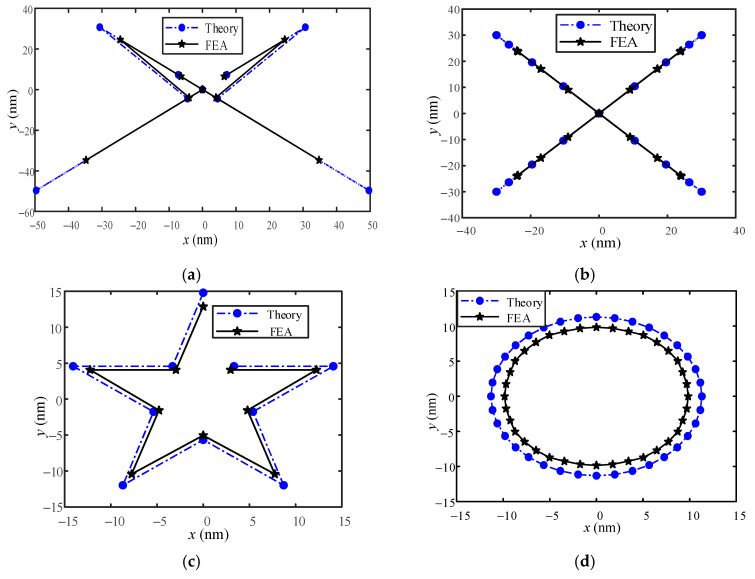
The coupled displacement of a five-pointed star and a circular trajectory. (**a**,**b**) are the coupled displacements in X- and Y-directions of the bottom platform, respectively. (**c**,**d**) are X- and Y-direction coupled displacements under Z-direction 100N load, respectively.

Next, the coupled displacement of the motion trajectory is compared and analyzed. This article studies that the coupled displacement in the motion trajectory of the positioning stage includes the coupled displacement between the X- and Y-direction motions and the X- and Y-direction coupled displacements under the influence of an external load in the Z-direction. Therefore, the five-pointed star and circular trajectory in the previous section are applied as references for analysis. The coordinates of the pentagram trajectory are shown in Table 5, and the circular trajectory is a circle with a radius of 60 µm. Since the dynamic resolution of the displacement sensor in the experiment is 100 nm and the environmental error in the measurement fluctuates within a few hundred nanometers, the experimental results are challenging to meet the accuracy requirements. The simulation results are used to verify the correctness of the theory. Figure 13a,b are the coupled displacement diagrams of the five-pointed star and the circular X- and Y-direction motions. From Figure 13a,b, it can be deduced that the theoretical results are consistent with the simulation results. Still, the error of the theoretical results increases with the increase of the coupled displacement. Figure 13c,d are the coupled displacements of X- and Y-directions under the influence of external load in the Z-direction, respectively. Figure 13c,d depict that the coupled displacement is related to the trajectory graph and varies in a particular proportion with the graph trajectory. The theoretical results are close to the FEA results.

**Figure 14:**
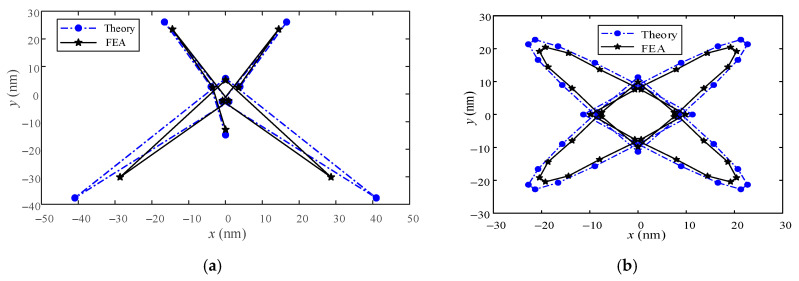
Comparison the theory and simulation about the combined coupling displacement. (**a**). The combined displacement diagram in the pentagram trajectory. (**b**) The combined displacement diagram in the circular trajectory.

Figure 14 is a comparison diagram of theory and simulation about the combined coupling displacement. Through Figure 14, it can be further determined that the study of the coupled displacement for the platform in this article is correct and conforms to the law of simulation analysis. Although it conforms to the law of change, the error is large, the maximum error is 42.86%, and the maximum stroke in the trajectory is 75 μm. If the motion stroke increases, the error of theoretical analysis will increase. The coupled displacement in the circular trajectory is very regular and symmetrical, which further confirms the above conclusion. The large deviation between the FEA results and the theoretical results, may be because the platform has other coupling motions in the motion, which is not considered in this paper.

**Figure 15:**
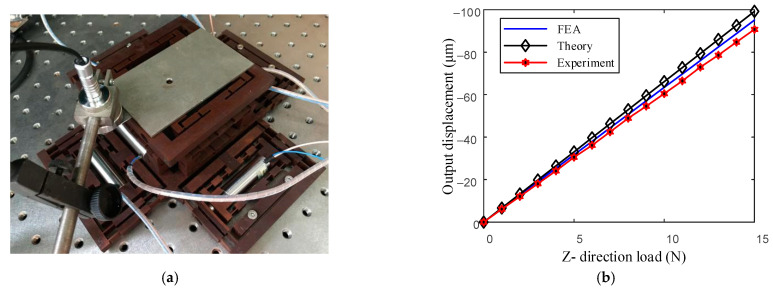
Comparison the theory and simulation of Z-direction displacement change under the influence of the Z-direction load. (**a**) Loading diagram of the positioning stage in the experiment. (**b**) Comparison of theory and simulation for displacement changes.

In addition, the effect of Z-direction load on displacement is analyzed. As shown in Figure 15a, the Z-direction load on the positioning stage is represented by placing aluminum blocks of the same mass on the upper surface of the top platform as the load reference surface, wherein the mass of each aluminum block is 100 g. The simulation, theoretical and experimental results are shown in Figure 15b when the positioning stage is subjected to the Z-direction load. From Figure 15b, the results are similar, and the average errors of the theoretical and experimental results relative to the simulation results are 4.34% and 3.69%, respectively. The above analysis shows that when the platform is loaded in the Z-direction, the theoretical analysis has a high prediction accuracy.

## 7. Conclusions

This paper discusses the applicability of the positioning stage based on the flexible hinge in noncontact processing. According to the actual application of the platform, the Euler–Bernoulli beam theory and the geometric relationship of the structure are used to analyze the coupling displacement of the platform X- and Y-direction motion and the changes in the performance and displacement of the platform caused by external loads such as workpieces and fixtures. The displacement expressions in the Z-direction and the coupling displacement expressions in X- and Y-directions under the load in Z-direction, and the coupling displacement expressions in the X- and Y-directions of the platform are obtained. Combine the above expressions to obtain the displacement expression of the platform in noncontact machining situations. The correctness of the theory is verified through experiments and simulation analysis.

This paper found that the coupling displacement of the X- and-Y direction is about 15 nm when Z-direction moves 0.5 mm under the action of the Z-direction load. It can be seen that the appropriate Z-direction load has little effect on the displacement of X- and Y-direction, which can meet the accuracy requirements of the trajectory in micron machining. When the X- and Y-direction displacements are 46 and 63 μm, the coupling displacement in the simulation analysis is about 30 nm, and the error is close to one-thousandth of the output displacement. The coupling displacement can be appropriately reduced by reducing the stiffness of the guiding mechanism and increasing the lateral stiffness of the BDA. In the range of PZA stroke, it can meet the accuracy requirements of trajectory in micron-level machining.

The analysis of this paper proves that the positioning stage based on a flexible hinge can meet the accuracy and stability requirements in noncontact machining. Subsequently, the trajectory accuracy of the positioning stage can be further improved through error compensation combined with the theory in this paper.

## Figures and Tables

**Table 1:** Comparison of developed micropositioning stages based on compliant mechanisms.

Stage	DOF	Motion Ranges	Coupling Ratio
[29]	2 (*XY*)	25 μm × 25 μm	Low
[30]	2 (*XY*)	181.0 μm × 179.5 μm	1.07%
[7]	3 (Z*θ_X_θ_Y_*)	190 μm, 0.5 μm × 0.5 mrad	Unknown
[13]	3 (*XYZ*)	177.33 μm × 179.30 μm × 17.45 μm	Less than 1.6%
[31]	3 (*XYθ*)	58.0 μm ×58.0 μm, 1.05 mrad	Less than 1.6%
[32]	6 (*XYZθ_X_θ_Y_θ_Z_*)	8.2 μm × 10.5 μm × 13.2 μm, 225 μrad × 107 μrad × 100 μrad	High
[2]	6 (*XYZθ_X_θ_Y_θ_Z_*)	291.80 μm × 297.42 μm × 442.65 μm, 9.22 mrad × 9.22 mrad × 2.71 mrad	XYZ: less than 0.1%, *θ_X_θ_Y_θ_Z_*: less than 3.08%

**Table 2:** Values of related parameters.

**Parameters**	***l* (mm)**	***l*_1_ (mm)**	***l_a_* (mm)**	***l_m_* (mm)**	***l_c_* (mm)**	***h* (mm)**	***b* (mm)**	***h*_1_ (mm)**
Values	8	15	1	9	14	0.8	10	5
Parameters	*h_m_* (mm)	*L* (mm)	*L_m_* (mm)	*L*_1_ (mm)	*L*_2_ (mm)	*b* (mm)	*b_t_* (mm)	*l*_2_ (mm)
Values	5	8	30	63	100	10	5	9
Parameters	*L*_3_ (mm)	*h_t_* (mm)	*E* (GPa)	ρ (kg/m^3^)	µ			
Values	95	10	206	7850	0.27			

**Table 3:** The specifications of PZA.

Properties	Values
Nominal displacement (μm)	38 ± 10%
Stiffness (N/μm)	60 ± 20%
Blocked force (N)	2300
Capacitance (μF)	7.2 ± 20%
Resonant frequency (kHz)	20
Dimensions (mm)	Φ15 × 50

**Table 4:** The comparative data of motion trajectory.

**FEA**	*z* (μm)	108.04	110.03	112.03	114.02	116.00	117.99	119.98	121.97	123.96	125.95
	*x* (μm)	−9.49	−18.67	−26.88	−33.51	−38.03	−40.05	−39.34	−35.87	−29.79	−21.43
**Theory**	*z* (μm)	112.49	114.57	116.64	118.71	120.78	122.85	124.92	126.99	129.06	131.14
	error (%)	4.12	4.13	4.11	4.11	4.12	4.12	4.12	4.12	4.11	4.12
	*x* (μm)	−9.99	−19.65	−28.30	−35.27	−40.03	−42.15	−41.41	−37.75	−31.35	−22.56
	error (%)	5.27	5.25	5.28	5.25	5.26	5.24	5.26	5.24	5.24	5.27
**Experiment**	*z* (μm)	106.87	109.57	111.34	113.44	115.50	116.61	119.63	121.06	123.36	125.32
	error (%)	1.08	0.42	0.62	0.50	0.43	1.17	0.29	0.74	0.48	0.50
	*x* (μm)	−9.42	−18.49	−26.75	−33.25	−37.89	−39.87	−38.96	−35.59	−29.49	−21.28
	error (%)	0.79	0.99	0.50	0.78	0.37	0.44	0.97	0.78	0.98	0.71

**Table 5:** Five-pointed star coordinate values.

Coordinate Point	1	2	3	4	5	6	7	8	9	10
***x*(μm)**	0	−17.634	−74.697	−28.532	−46.165	0	46.165	28.532	74.697	17.634
***y*(μm)**	78.541	24.271	24.271	−9.271	−63.541	−30	−63.541	−9.271	24.271	24.271
